# Supplementary material for: New Protocol for Production of Reduced-Gluten Wheat Bread and Pasta and Clinical Effect in Patients with Irritable Bowel Syndrome: A randomised, Double-Blind, Cross-Over Study
Source: Nutrients. 2018 Dec 2;10(12):1873. doi: 10.3390/nu10121873 (PMC6316462; doi:10.3390/nu10121873)
Supplement: Supplementary file 1 [file nutrients-10-01873-s001.pdf]

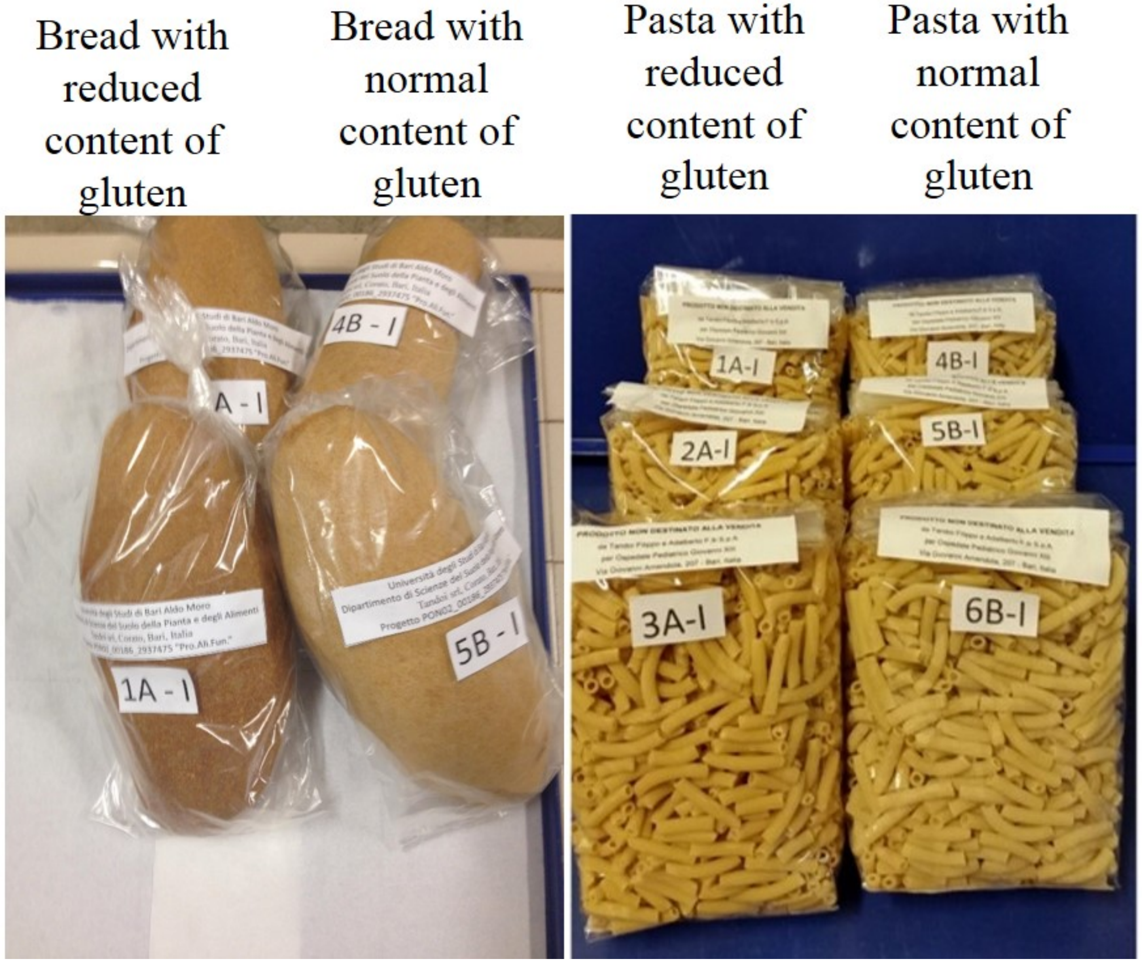

Figure S1. Bread and pasta made with normal and reduced content of gluten.

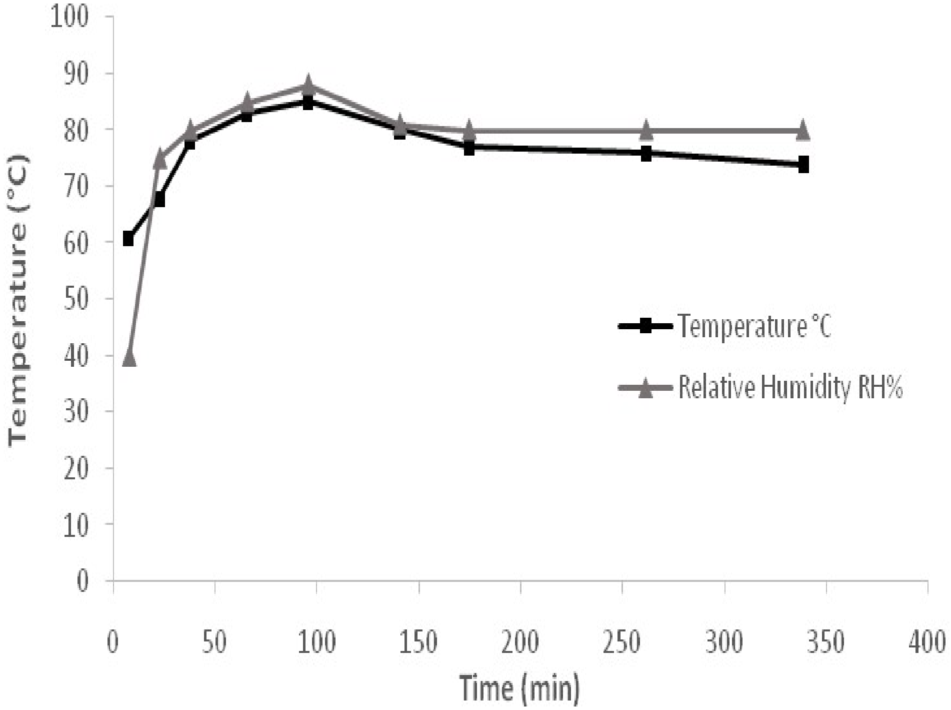

Figure S2. Dynamic drying cycle at medium-high temperatures used for making pasta.

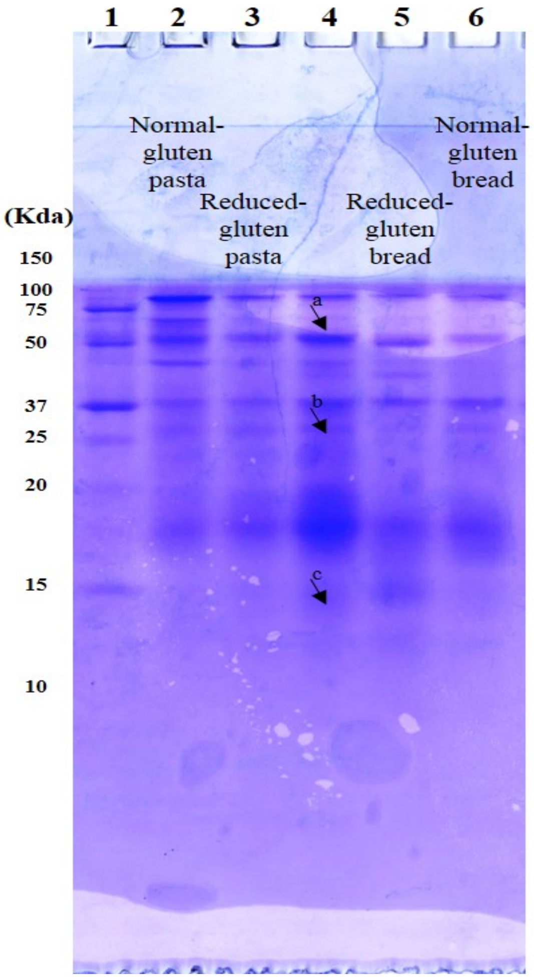

Figure S3. SDS-PAGE of albumins from bread and pasta made with normal- and reduced-gluten content flours. Reduced-gluten wheat flour was fermented with fungal proteases and selected lactobacilli at 30°C for 8 h. Lane 1: protein molecular weight standard; Lane 2: albumins extract from pasta made with normal-gluten content flours; Lane 3: albumins extract from pasta made with reduced-gluten content flours; Lane 4: albumins extract from not-fermented dough made with normal-gluten content flours; Lane 5: albumins extract from bread made with reduced-gluten content flours; Lane 6: albumins extract from bread made with normal-gluten content flours. Arrows indicate α-amylase/trypsin inhibitors (ATIs) in different forms: a, tetramer (60 kDa); b, dimer (24 kDa) and c, monomer (12 kDa). kDa: kilodalton.

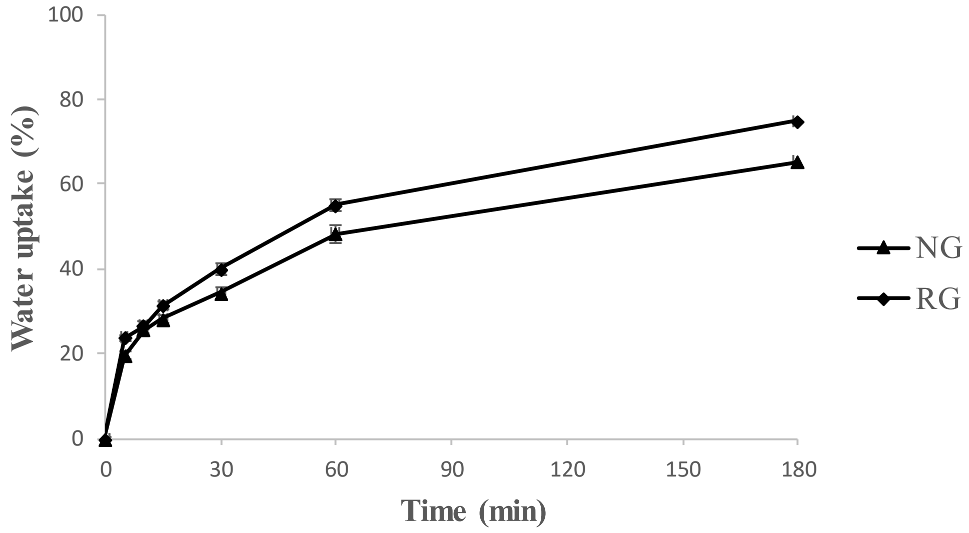

Figure S4. Kinetics of water absorption of pasta made with normal (NG) and reduced content of gluten (RG) flours, at 25 °C. RG wheat flour was fermented with fungal proteases and selected lactobacilli at 30°C for 8 h. Data are the means of three independent analyses. Bars of standard deviations are also represented.

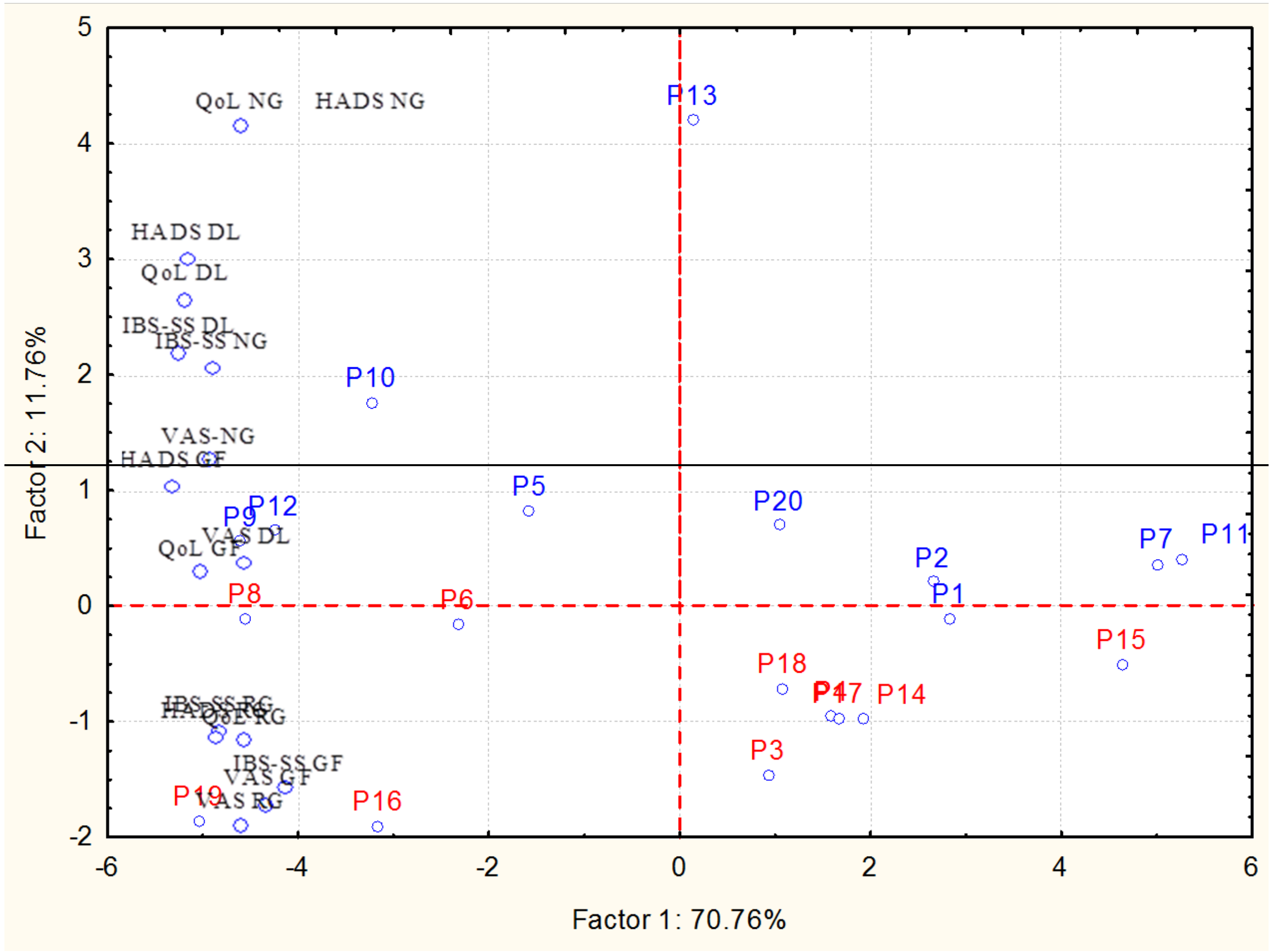

Figure S5. Score and loading plots of first and second principal components after principal component analysis based on clinical scores. Cases (patients) were numbered from P1 to P20. Variables were reported as abbreviation of clinical scores (IBS-SS, Irritable Bowel Syndrome Severity Score; VAS, Visual Analogue Scale; HADS, Hospital Anxiety and Depression Scale; QoL, Quality of Life) plus the type of diet (DL, Gluten Containing Diet; GF, Gluten Free diet; RG, Reduced Gluten diet; NG, Normal Gluten Diet).

Table S1. Baseline demographic and clinical characteristics[1] of the enrolled patients.

Patient code	Sex	Age	BSS	IBS-SS	VAS	HADS	QoL	Asthenia	Headache	Mouthulcers
P1	F	33	IBS-M	100	2.7	7	15	2	2	0
P2	M	43	IBS-M	205	4.2	14	30	2	0	0
P3	M	36	IBS-D	210	4.3	14	33	2	0	1
P4	M	45	IBS-D	195	3.5	12	27	3	2	0
P5	F	44	IBS-M	280	4.0	30	70	1	0	0
P6	F	46	IBS-D	250	3.4	33	89	1	1	0
P7	F	31	IBS-M	125	3.2	8	15	1	3	0
P8	M	47	IBS-M	310	5.1	39	89	3	0	0
P9	F	63	IBS-C	330	5.2	40	88	1	0	0
P10	M	19	IBS-D	330	4.9	39	80	1	1	0
P11	M	20	IBS-C	140	2.5	10	22	3	3	0
P12	F	65	IBS-M	300	7.0	38	80	0	3	0
P13	F	31	IBS-M	300	3.4	38	77	0	1	0
P14	M	27	IBS-M	180	2.3	11	25	3	2	1
P15	F	33	IBS-D	200	1.9	11	26	1	3	0
P16	F	55	IBS-C	305	7.0	29	66	0	3	0
P17	F	39	IBS-C	220	2.4	13	30	2	0	0
P18	F	27	IBS-M	215	2.3	14	33	1	1	0
P19	M	66	IBS-D	320	6.2	38	78	0	0	0
P20	F	23	IBS-D	230	4.5	21	43	0	0	0

[1]F, Female; M, Male; BSS, Bristol Stool Charts; IBS-SS, Irritable Bowel Syndrome Severity Score; VAS, Visual Analogue Scale; HADS, Hospital Anxiety and Depression Scale; QoL, Quality of Life. IBS-C, constipation predominant; IBS-D, diarrhea-predominant; IBS-M, mixed diarrhea/constipation.

Table S2 Modification of the clinical scores[1] in the responders and non responders irritable bowel syndrome (IBS) patients in the different diets according to the challenge results.

Clinical scores	Diet	Responders (n = 10) Average value ± SD	Non responders (n = 10) Average value ± SD	Clinical scores	Habitual diet *vs* Run-in Open gluten-free diet	Run-in Open gluten-free diet vs. Reduced-gluten diet	Run-in Open-gluten-free diet vs. Normal-gluten diet	Reduced-gluten diet vs Normal-gluten diet
					**Responders (n = 10) P value**			
**IBS-SS**	Run-in Open gluten-free diet	128.0 ± 77.82	201.5 ± 64.55	**IBS-SS**	0.001	0.059	0.002	0.048
	Reduced-gluten diet	176.4 ± 119.12	252.5 ± 51.92	**VAS**	0.000	0.200	0.001	0.015
	Normal-gluten diet	223.5 ± 96.73	235.5 ± 63.35	**HADS**	0.005	0.033	0.016	0.014
**VAS**	Habitual diet	4.2 ± 1.34	3.9 ± 1.77	**QoL**	0.000	0.000	0.007	0.009
	Run-in Open gluten-free diet	2.5 ± 1.16	3.2 ± 1.60					
	Reduced-gluten diet	2.9 ± 1.78	4.1 ± 1.44					
	Normal-gluten diet	4.3 ± 1.65	4.2 ± 1.77					
**HADS**	Habitual diet	24.5 ± 13.97	21.4 ± 11.84		**Non responders (n = 10) P value**			
	Run-in Open gluten-free diet	18.6 ± 11.63	22.0 ± 9.90	**IBS-SS**	0.005	0.002	0.038	0.159
	Reduced-gluten diet	15.0 ± 12.11	24.4 ± 7.18	**VAS**	0.000	0.000	0.007	0.091
	Normal-gluten diet	23.7 ± 15.00	21.6 ± 8.73	**HADS**	0.312	0.032	0.029	0.102
**QoL**	Habitual diet	52.0 ± 29.84	49.6 ± 27.46	**QoL**	0.042	0.007	0.001	0.280
	Run-in Open gluten-free diet	34.9 ± 24.74	44.0 ± 25.55					
	Reduced-gluten diet	32.8 ± 27.44	57.5 ± 19.57					
	Normal-gluten diet	53.4 ± 30.00	41.2 ± 17.47					

[1]IBS-SS, Irritable Bowel Syndrome Severity Score; VAS, Visual Analogue Scale; HADS, Hospital Anxiety and Depression Scale; QoL, Quality of Life.
